# Cord Blood Serum Attenuates Hyperosmolarity-Induced Inflammation and TRPV1 Upregulation in Conjunctival Epithelial Cells

**DOI:** 10.3390/ijms27031290

**Published:** 2026-01-28

**Authors:** Gloria Astolfi, Carmen Ciavarella, Chiara Coslovi, Elisa Bergantin, Marina Buzzi, Luigi Fontana, Piera Versura

**Affiliations:** 1 Ophthalmology Unit, DIMEC, Alma Mater Studiorum Università di Bologna, 40138 Bologna, Italy; gloria.astolfi2@unibo.it (G.A.); carmen.ciavarella2@unibo.it (C.C.); chiara.coslovi@unibo.it (C.C.); luigi.fontana6@unibo.it (L.F.); 2 IRCCS Azienda Ospedaliero-Universitaria di Bologna, 40138 Bologna, Italy; 3 Emilia Romagna Cord Blood Bank, IRCCS Azienda Ospedaliero-Universitaria di Bologna, 40138 Bologna, Italy; elisa.bergantin@aosp.bo.it (E.B.); marina.buzzi@aosp.bo.it (M.B.)

**Keywords:** hyperosmolar stress, conjunctival epithelial cells, cord blood serum, TRPV1 nociception, inflammation, HLA-DR, pain modulation

## Abstract

Eye drops derived from human blood components (Eye Drops of Human Origin—EDHO) have proven effective in reducing ocular pain associated with severe keratopathies. Among these, Cord Blood Serum (CBS) is particularly promising for its high content of growth and neurotrophic factors. This study evaluated the ability of CBS to modulate inflammatory and nociceptive activation in the human conjunctival epithelial cell (HCEC) line exposed to hyperosmotic stress. CBS batches were characterized for brain-derived neurotrophic factor (BDNF) content and classified as CBS_high_ (levels > 18.0 ng/mL) or CBS_low_ (levels < 10.0 ng/mL). HCECs were exposed to NaCl (450 mOsm/L) with or without 5% CBS. Cell viability was evaluated, and the expression of Major Histocompatibility Complex Class II (HLA-DR) (a marker of immune activation) and Transient Receptor Potential Vanilloid 1 (TRPV1) (a nociceptive ion channel responsive to osmotic stress) was assessed via Real Time PCR (RT-PCR). CBS significantly improved HCEC viability under hyperosmotic stress. Exposure to NaCl alone upregulated *HLA-DR* and *TRPV-1* expression. Both CBS preparations attenuated these responses, producing comparable reductions in HLA-DR mRNA and decreasing TRPV-1 expression. Partial reversal of CBS effects by the pan-neurotrophin receptor inhibitor K252a supported neurotrophin involvement. CBS reduces hyperosmolarity-driven inflammation and nociception via HLA-DR and TRPV1 downregulation, supporting its role as a bioactive tear substitute in neuroinflammatory ocular surface disease.

## 1. Introduction

Dry eye disease (DED) is a multifactorial disorder of the ocular surface, characterized by a loss of tear film homeostasis and accompanied by ocular symptoms such as discomfort and visual disturbance, in which tear film instability and hyperosmolarity, ocular surface inflammation and damage, and neurosensory abnormalities play etiological roles [1]. It is recognized as a chronic and relapsing condition with a significant impact on quality of life and visual function [2,3]. Clinical management remains challenging, in part due to the frequent poor correlation between the severity of patient-reported symptoms and objective clinical signs, reflecting the complex and heterogeneous nature of DED pathophysiology [1,4].

From the patient’s perspective, relief from ocular pain and irritation is often the foremost therapeutic goal, as these symptoms substantially impair quality of life and daily activities [2,5].

Recently, cord blood serum (CBS) has gained attention as a promising therapeutic option for a range of ocular surface diseases, including DED and neuropathic corneal pain (NCP). Compared to other blood-derived eye drops, CBS is enriched with higher concentrations of epithelial and neurotrophic growth factors (NTFs), such as epidermal growth factor (EGF), nerve growth factor (NGF), insulin-like growth factor-1 (IGF-1), transforming growth factor-beta (TGF-β), and vascular endothelial growth factor (VEGF), which are critical for epithelial healing, modulation of inflammation, and corneal nerve regeneration [6]. Several studies showed ocular surface subjective pain reduction after treatment with blood-based products [7].

Several preclinical and clinical studies have demonstrated the neuroregenerative potential of CBS, with improvements in sub-basal nerve density, length, and morphology, as well as reductions in nerve tortuosity, beading, and neuromas, as assessed by in vivo confocal microscopy [8]. These morphological changes correlate with significant symptom relief, including reduced corneal pain, photophobia, and other pain-like sensations [9].

The purpose of this work was to explore the anti-inflammatory potential of cord blood serum (CBS) and its capability to modulate Transient Receptor Potential Vanilloid 1 (TRPV1) expression in human conjunctival epithelial cells exposed to hyperosmolar stress. An in vitro model was selected to enable the controlled investigation of epithelial-specific responses to tear film hyperosmolarity, a well-established core mechanism in dry eye disease (DED) pathophysiology [1,10]. Hyperosmolar stress is known to trigger both inflammatory and vanilloid channel expression at the ocular surface, including the upregulation of key biomarkers such as Major Histocompatibility Complex Class II (MHC II) (HLA-DR), a marker of immune activation [11,12,13], and TRPV1, an ion channel expressed in conjunctival epithelial cells [14], and implicated in neurogenic inflammation in DED [8,15]. Nociception, as defined by the International Association for the Study of Pain (IASP), is the neural encoding of noxious stimuli and depends on sensory neuron activity. We therefore focused on epithelial responses involving TRPV1, a channel known to contribute to both nociceptive and neuroinflammatory processes [16].

Human conjunctival epithelial cells were specifically chosen because the conjunctiva constitutes the exposed mucosal surface of the ocular surface system, playing a critical role in sensing and responding to environmental and osmotic stressors [17], and its mucosal nature makes it particularly relevant for studying epithelial inflammation [18]. This model provides a relevant platform to dissect early molecular events and assess the therapeutic potential of CBS in modulating cellular mechanisms relevant to dry eye disease.

## 2. Results

### 2.1. CBS Counteracts the Hyperosmolar-Induced Cell Damage

**Figure 1:**
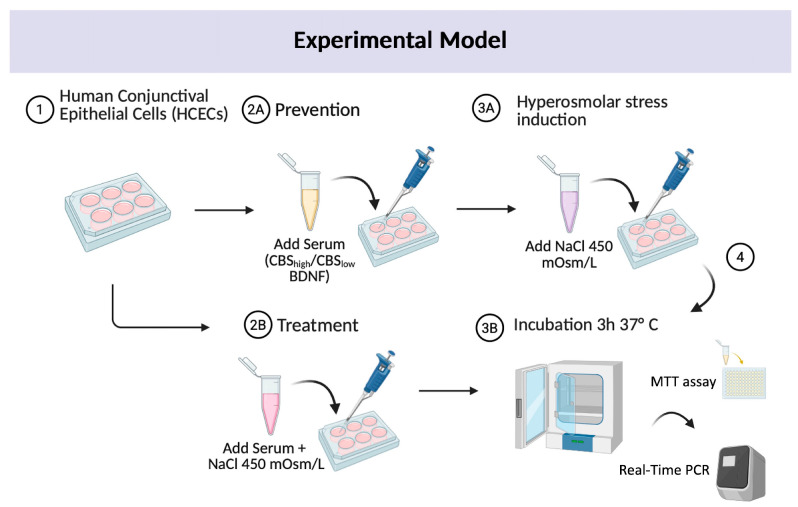
Experimental design representation. This workflow represents an experimental model for investigating the role of CBS in modulating induced hyperosmolar stress. (1) HCECs were seeded and (2A) pre-treated with BCS/CBS_low_/CBS_high_ prior to (3A) hyperosmotic exposure or (2B) co-cultured with BCS/CBS_low_/CBS_high_ during NaCl induction. After (3B) incubation, (4) cell viability assay was performed, and molecular expression was evaluated. Created in BioRender. Astolfi, G. (2025). https://biorender.com (accessed on 14 October 2025).

CCL-20.2 cells were exposed to hyperosmolar stress induced by NaCl (450 mOsm), a central condition in the pathophysiology of dry eye disease. To assess the effect of CBS with different BDNF contents, cells were either pre-treated with CBS prior to NaCl exposure or co-cultured with CBS during NaCl treatment (CBS + NaCl). The experimental design is represented in Figure 1.

**Figure 2:**
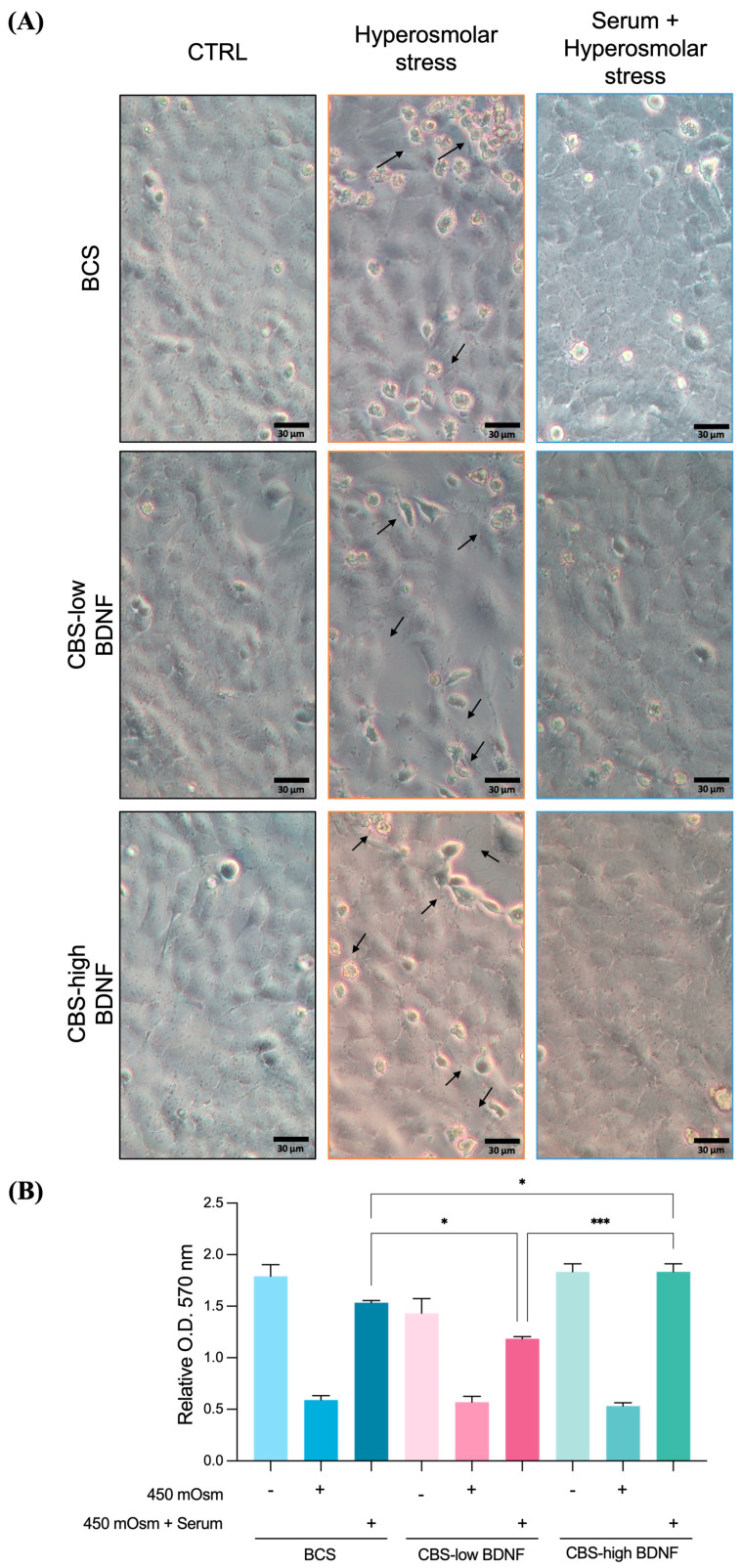
CBS administration in combination with NaCl prevented cellular injury and mortality. Analysis of (**A**) cell morphology and (**B**) cell viability, measured by MTT assay, under NaCl administration alone or in combination with serum (BCS/CBS_low_/CBS_high_). Treated and non-treated groups are clearly distinguished, and “+/−” symbols are displayed below each bar to indicate the presence or absence of the treatment conditions. Arrows indicate cell injury. Experimental groups: BCS (CTRL: untreated control; 450 mOsm: cells exposed to NaCl in serum-free medium; 450 mOsm + Serum: cells exposed to NaCl in BCS complete medium; CBS_low_ (CTRL: untreated control; NaCl: cells exposed to NaCl in serum-free medium; 450 mOsm + Serum: cells exposed to NaCl in CBS_low_ complete medium; CBS_high_ (CTRL: untreated control; NaCl: cells exposed to NaCl in serum-free medium; 450 mOsm + Serum: cells exposed to NaCl in CBS_high_ complete medium). MTT results are shown as relative absorbance measured at an optical density (O.D.) of 570 nm and are reported as the mean ± standard deviation of three independent experiments. *, *p* < 0.05; ***, *p* < 0.001 (Ordinary one-way ANOVA, followed by Tukey’s multiple comparison tests).

As expected, NaCl exposure for 3 h reduced cell viability, accompanied by early morphological alterations and detachment indicated with arrows (Figure 2A). No protective effect on cell viability was observed when CBS was administered as a pretreatment before the NaCl challenge. In contrast, co-treatment with CBS_high_ effectively preserved cell morphology (Figure 2A) and viability, as confirmed by the MTT assay results (Figure 2B).

### 2.2. CBS Modulates NaCl-Induced Inflammatory Injury Through Regulation of HLA-DR Expression

**Figure 3:**
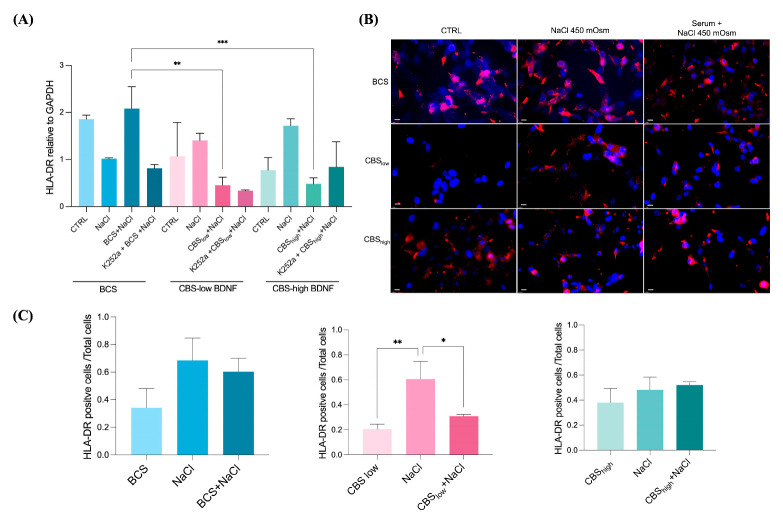
Protective and mitigating effects of CBS on the inflammatory marker HLA-DR in CCL20.2 cells exposed to hyperosmolar stress. (**A**) HLA-DR mRNA levels were investigated using real-time PCR. HLA-DR Ct values were normalized to the GAPDH gene, and the final data are reported as fold relative to untreated controls and mean ± standard deviation of three biological replicates. (**B**) HLA-DR protein analyzed by immunofluorescence and (**C**) relative quantification performed by measuring the ratio of HLA-DR positive cells to total cells for three fields randomly selected at 20× magnification. Red: HLA-DR, blue: dapi. Scale bar: 10 μm, 40× magnification. Experimental groups: BCS (CTRL: untreated control; NaCl: cells exposed to NaCl in serum-free medium; BCS+NaCl: cells exposed to NaCl in BCS complete medium; K252a + BCS + NaCl: cells pre-treated with K252a, then exposed to NaCl in BCS complete medium); CBS_low_ (CTRL: untreated control; NaCl: cells exposed to NaCl in serum-free medium; CBS_low_ + NaCl: cells exposed to NaCl in CBS_low_ complete medium; K252a + CBS_low_ + NaCl: cells pre-treated with K252a, then exposed to NaCl in CBS_low_ complete medium); CBS_high_ (CTRL: untreated control; NaCl: cells exposed to NaCl in serum-free medium; CBS_high_ + NaCl: cells exposed to NaCl in CBS_high_ complete medium, K252a + CBS_high_ + NaCl: cells pre-treated with K252a, then exposed to NaCl in CBS_high_ complete medium). Results are shown as mean ± standard deviation (SD) of three biological replicates. *, *p* < 0.05; **, *p* < 0.01; ***, *p* < 0.001. (Ordinary one-way Anova, followed by Tukey’s multiple comparison tests applied to all panels).

Given the leading role of inflammation in the pathogenesis of dry eye disease, we evaluated whether CBS could influence HLA-DR expression, a recognized inflammatory marker. As shown in Figure 3A, co-administration of CBS and NaCl significantly reduced HLA-DR transcript levels at both BDNF concentrations. To assess the contribution of BDNF, the cells were treated with K252a, a TrkB inhibitor. As explicated in the Section 4 of this article, the concentration of K252a was selected based on our previous study, where it effectively inhibited Trk-mediated neurotrophin signaling without inducing cytotoxicity [19]. Regarding the experimental design, K252a was applied only as a pre-incubation step; transient inhibition is the most appropriate strategy to assess the contribution of BDNF/Trk signaling to subsequent treatment. Furthermore, continuous exposure during CBS incubation could cause a sustained and complete Trk blockade, potentially masking or abolishing CBS-mediated effects and preventing the evaluation of its neurotrophin-dependent activity. Pre-incubation, therefore, ensures that Trk is inhibited at the time of CBS administration while still allowing detection of any residual or alternative CBS effects beyond Trk signaling. Inhibition with K252a partially reversed the protective effect of CBS_high_, resulting in increased HLA-DR mRNA expression (Figure 3A). The anti-inflammatory action of CBS during hyperosmolar stress was further confirmed by immunofluorescence, which demonstrated a marked reduction in HLA-DR signal, particularly in cells treated with CBS_low_ (Figure 3B,C). Notably, a decrease in HLA-DR–positive cells was also observed when CBS was applied as a pretreatment prior to NaCl exposure (Figure 3B,C).

### 2.3. CBS Modulates TRPV-1 Expression

**Figure 4:**
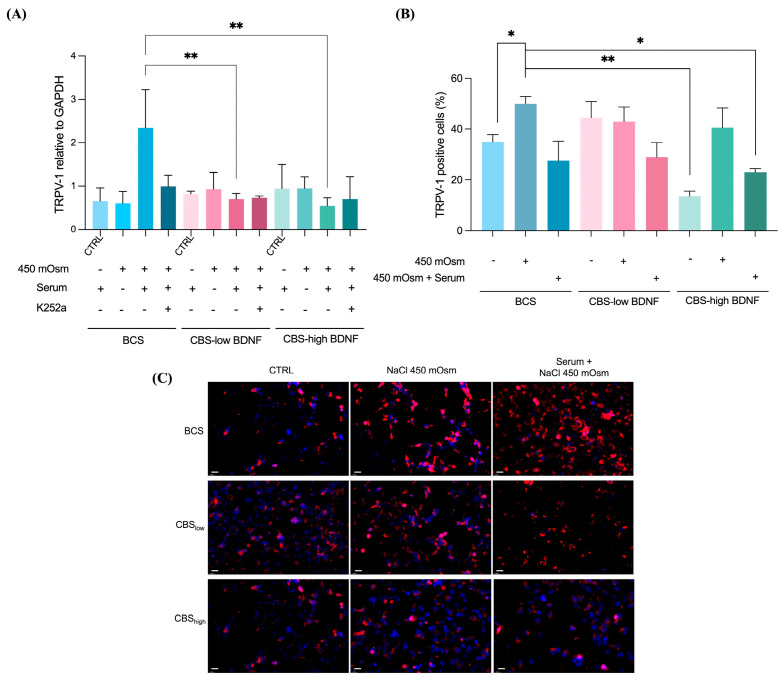
TRPV-1 expression in human conjunctival cells. (**A**) *TRPV-1* mRNA expression following NaCl hyperosmolar stress. (**B**) Detection and quantification of TRPV1 positive cells using Countess 3 FL. Results are shown as mean ± SD of three biological replicates. “+/−” symbols displayed below each bar indicate the presence or absence of the treatment conditions. *, *p* < 0.05; **, *p* < 0.01. Experimental groups for each Serum (BCS/CBS_low_/CBS_high_): CTRL: untreated control; 450 mOsm: cells exposed to NaCl in serum-free medium; K252a: cells pre-treated with K252a, then exposed to NaCl 450 mOsm in medium supplemented with serum. Statistical analysis was performed by a one-way ordinary ANOVA test followed by a Tukey post hoc test for multiple comparisons. (**C**) Immunofluorescence analysis of TRPV-1. Blue: nuclei; red: TRPV-1. Scale bar 25 μm; 20× magnification.

The expression of TRPV1 protein was markedly reduced in cells cultured with CBS_high_ compared to those treated with BCS, indicating a stronger regulatory effect on nociceptive signaling. When combined with NaCl, CBS_high_ further decreased *TRPV-1* expression and significantly reduced the proportion of TRPV1-positive cells (Figure 4A–C). This effect was less pronounced in CBS_low_ cells, suggesting that a higher BDNF content contributes to the modulation of TRPV1 activity under hyperosmolar stress. Consistently, cells treated with CBS_high_ maintained a more stable morphology and exhibited reduced signs of stress-related hyperexcitability, supporting the role of CBS in attenuating nociceptor sensitization through the downregulation of TRPV1. Notably, as BDNF can influence TRPV1 activity through TrkB-dependent signaling pathways, these findings suggest that the elevated BDNF content in CBS_high_ may contribute to the attenuation of nociceptive responses through modulation of TRPV1 activity and prevention of its hyperactivation under stress conditions.

## 3. Discussion

In this study, we demonstrated that CBS exerts a dual protective effect against hyperosmolarity-induced injury in human conjunctival epithelial cells, attenuating both inflammatory and TRPV1 expression. Specifically, CBS significantly reduced the expression of HLA-DR, a recognized marker of immune activation in dry eye disease, and TRPV1, a transient receptor potential channel present also in conjunctival epithelial cells, which may represent a proxy for nociceptive-related signaling. These results support the hypothesis that ocular surface inflammation and pain are tightly interconnected through shared molecular pathways, and that CBS may act as a multimodal modulator targeting this neuro-immune interface. The preservation of cell viability and morphology under hyperosmotic stress further indicates that CBS not only mitigates cellular stress responses but also promotes epithelial homeostasis. In the interpretation of cell viability outcomes assessed by the MTT assay, we chose to perform a combined analysis of the experimental groups rather than evaluating each condition in isolation. This strategy was adopted to enable a direct comparison between the effects of CBS and the reference condition (BCS) within the same experimental context. By analyzing the data comparatively, we aimed to minimize inter-assay variability and to better contextualize the relative impact of CBS on cellular metabolic activity under hyperosmolar stress. In this context, evaluating all experimental conditions within a unified analytical model increases statistical power and allows differences between treatments to be more readily identified, particularly in comparative in vitro studies in which multiple interventions are assessed under shared injury conditions. Importantly, although the data were analyzed within a common framework, each experimental condition (control, NaCl, and serum + NaCl) was treated as an independent level within the model, thereby preserving appropriate statistical assumptions and ensuring the validity of the comparisons. Taken together, this approach supports a robust and integrative interpretation of the viability data in relation to the morphological and molecular findings discussed above.

When the Trk inhibitor is applied to cells treated with CBS_low_, HLA-DR expression does not increase. Given that the main difference between CBS_low_ and CBS_high_ lies in their BDNF content, modulation of Trk receptor signaling is expected to differentially influence CBS-mediated protection. In CBS_high_, higher BDNF levels confer a greater reliance on Trk-dependent pathways, such that Trk inhibition results in a marked reduction in CBS protective effects, as evidenced by increased HLA-DR expression. In contrast, the lower BDNF content of CBS_low_ is associated with limited Trk activation, and its pharmacological inhibition does not substantially alter the inflammatory response. Overall, these findings indicate that CBS-mediated protection is at least partially driven by neurotrophin-dependent mechanisms, as supported by the attenuation of protective effects following Trk receptor inhibition with K252a. Pre-incubation therefore ensures that Trk are inhibited at the time of CBS administration, while still allowing detection of any residual or alternative CBS effects beyond Trk signaling. Taken together, this suggests that growth factors such as BDNF, abundantly present in CBS [20], contribute to the restoration of epithelial resilience through both trophic and anti-inflammatory mechanisms. Collectively, our findings position CBS as a biologically active tear substitute capable of modulating the bidirectional interaction between inflammation and nociception that underlies ocular surface discomfort and neuropathic pain [4,5].

Hyperosmolarity is a well-established trigger of ocular surface inflammation, leading to the activation of stress pathways and upregulation of HLA-DR expression in conjunctival epithelial cells [11,17]. We recently demonstrated the up-regulation of HLA-DR protein in CCL-20.2 cells under hyperosmolar stress [21]. HLA-DR, a class II major histocompatibility complex molecule, is a hallmark of epithelial immune activation and reflects the engagement of the local antigen-presenting machinery [17]. In our model, hyperosmotic challenge induced a robust increase in HLA-DR transcripts, confirming epithelial activation under desiccating stress. The co-administration of CBS significantly suppressed this upregulation, supporting its capacity to dampen epithelial immune activation.

In the present study, the reduction in HLA-DR expression suggests that CBS can restore epithelial immune quiescence by modulating intracellular stress signaling. Consistent with a neurotrophin-dependent mechanism, BDNF/TrkB signaling has been shown to attenuate NF-κB activation in ocular surface epithelium under desiccating stress and to functionally interact within TRPV1–TrkB regulatory axis [22]. This may support a plausible route by which CBS may mitigate both inflammatory and nociceptive responses. This interpretation is in line with our previous findings demonstrating a coordinated upregulation of HLA-DR and TRPV1 in conjunctival epithelial cells exposed to hyperosmotic stress [11], and with broader evidence of BDNF’s anti-inflammatory actions in neural and epithelial systems.

The observation that CBS_low_ exerted a comparable anti-inflammatory effect to CBS_high_, despite lower BDNF levels, indicates that non-neurotrophin components may play a dominant role in controlling epithelial inflammation. This supports a multifactorial mechanism of CBS action, in which diverse trophic and immunomodulatory factors synergistically preserve epithelial homeostasis.

Importantly, the suppression of HLA-DR expression in conjunctival epithelium has been associated with clinical improvement in dry eye disease and with the recovery of ocular surface integrity [23,24]. Therefore, the ability of CBS to downregulate this biomarker reinforces its therapeutic potential in inflammatory keratopathies characterized by chronic immune activation.

The transient receptor potential vanilloid 1 (TRPV1) channel is a polymodal nociceptor activated by osmotic, chemical, and thermal stimuli, and plays a pivotal role in ocular pain perception and neurogenic inflammation [8,15]. In ocular surface epithelial cells, hyperosmolar stress has been shown to activate and upregulate TRPV1, leading to calcium influx and stimulation of pro-inflammatory signaling pathways. TRPV1 activation contributes to the release of inflammatory mediators and neuropeptides such as substance P and calcitonin gene-related peptide (CGRP), promoting neurogenic inflammation and nociceptor sensitization [25]. This process contributes to peripheral sensitization and the maintenance of chronic ocular discomfort in dry eye disease.

In the present study, hyperosmolar exposure markedly increased TRPV1 expression in conjunctival epithelial cells. Notably, as BDNF can influence TRPV1 activity through TrkB-dependent signaling pathways, these findings suggest that the elevated BDNF content in CBS_high_ may contribute to the attenuation of nociceptive responses through modulation of TRPV1 activity and prevention of its hyperactivation under stress conditions [26]. The attenuation of TRPV1 activation by CBS suggests a direct effect on epithelial nociceptive signaling, consistent with its previously reported neuroprotective actions on corneal nerves [27]. The stronger downregulation observed with CBS_high_ supports the contribution of neurotrophins, particularly BDNF and NGF, which modulate TRPV1 phosphorylation and trafficking through TrkB-dependent pathways [28]. Indeed, pharmacological inhibition with K252a partially reversed the CBS-mediated reduction in TRPV1, confirming the involvement of neurotrophin signaling in the nociceptive modulation. These findings indicate that CBS can suppress epithelial TRPV1 overactivation, thereby reducing nociceptor sensitization and the subsequent neurogenic inflammatory feedback. This desensitizing effect may contribute to the symptomatic relief reported in patients treated with blood-derived eye drops and supports the use of CBS as a potential therapy targeting both inflammatory and neuronal pathways underlying ocular pain.

As already quoted, in a previous study, we demonstrated a functional association between HLA-DR and TRPV1 expression in human conjunctival epithelial cells, suggesting that inflammatory and nociceptive pathways may be co-regulated under stress conditions [11]. Although a molecular interaction between HLA-DR and TRPV1 has not been shown in the present study as well, the concurrent downregulation of these two markers following CBS exposure further supports the hypothesis of a functional crosstalk between epithelial immune and sensory pathways. Increasing evidence indicates that chronic ocular surface inflammation and nociceptive sensitization are mutually reinforcing processes, sustained by shared mediators such as cytokines, neuropeptides, and reactive oxygen species [5,17]. Hyperosmolar stress has been shown to promote the release of inflammatory cytokines (IL-6, TNF-α, IL-1β) that can sensitize TRPV1 channels, whereas TRPV1 activation itself enhances epithelial secretion of pro-inflammatory factors, thereby amplifying local immune activation [8].

Within this framework, CBS may act as a dual modulator capable of interrupting this vicious cycle by simultaneously reducing epithelial immune activation and nociceptor over-excitability. The downregulation of HLA-DR could decrease antigen presentation and T-cell recruitment, while TRPV1 inhibition may limit neuropeptide-driven inflammation. Together, these effects are consistent with a shift toward epithelial homeostasis and decreased neurogenic inflammation at the ocular surface.

Nevertheless, several limitations should be acknowledged. The current model is restricted to epithelial monocultures and therefore does not fully reproduce the complex neuroimmune interactions occurring in vivo, where immune cells and sensory neurons dynamically influence each other. Moreover, CBS composition is inherently variable among donors, and the contribution of other substances or growth factors beyond BDNF to its overall bioactivity warrants further investigation.

Future studies should aim to integrate epithelial and neuronal cell models or employ organotypic cultures to better elucidate the crosstalk between CBS-mediated trophic signals, nociceptor modulation, and inflammatory control. Such approaches will help clarify the translational potential of CBS as a neuroprotective and anti-inflammatory tear substitute for chronic ocular pain and advanced dry eye disease.

## 4. Materials and Methods

### 4.1. Cell Culture

The present study was performed on a human conjunctival cell line (Wong Kilbourne derivative of Chang; clone 1-5c-4), obtained from the American Type Culture Collection (ATCC) (CCL-20.2; Manassas, VA, USA) and expanded in culture medium 199 (Gibco, Thermo Fisher Scientific, Waltham, MA, USA) supplemented with 10% bovine calf serum (BCS) ion-fortified obtained from ATCC (Manassas, VA, USA) and 1% penicillin (10,000 U/mL)/streptomycin (10,000 μg/mL, Lonza Group Ltd., Basel, Switzerland). Cells were grown in culture flasks at 37 °C in a humidified atmosphere with 5% CO_2_, and the media were changed every 2–3 days.

### 4.2. Cord Blood Serum (CBS) Preparation

CB collection and processing were performed in accordance with the Foundation for the Accreditation of Cellular Therapy (FACT) standards and national regulations, as previously described [20]. Briefly, CB was collected in utero immediately after delivery using a sterile closed collection system (JMS, Singapore) containing citrate–phosphate–dextrose (CPD) as anticoagulant. For serum preparation, additional samples were obtained ex utero from placental vessels into vacuum tubes (Biomed Device, Modena, Italy) without anticoagulant and transported to the accredited Processing Facility within 48 h. CB units were derived from healthy term deliveries (≥37 weeks of gestation) after maternal screening for infectious diseases (HIV, HBV, HCV, Treponema pallidum, CMV, Toxoplasma gondii, and HTLV-I/II). Hematological parameters and blood group typing were recorded to confirm the suitability of the units. Samples collected without anticoagulants were allowed to clot for 24–36 h at 4 °C and then centrifuged at 3500× *g* for 10 min. The supernatant serum (SS) was carefully aspirated, aliquoted, and stored at −80 °C until use.

#### BDNF Levels in CBS

All CBS batches were quantitatively characterized for BDNF content prior to biological testing. BDNF levels showed marked inter-batch variability and a non-Gaussian distribution, with the presence of distinct low- and high-concentration ranges rather than a continuous spectrum. BDNF levels exhibited high interindividual variability; therefore, based on this empirical distribution, CBS preparations were stratified into two non-overlapping groups according to BDNF concentration, defined as CBS_low_ (6–10 ng/mL) and CBS_high_ (18–23 ng/mL) groups. These groups were used for experimental comparisons, while samples with intermediate BDNF values were excluded to avoid overlap and misclassification. This conservative, data-driven stratification was applied consistently across all experiments and defined prior to downstream analyses. BDNF concentrations were measured in CBS samples using a commercial ELISA kit (Quantikine ELISA; R&D Systems, Minneapolis, MN, USA) according to the manufacturer’s instructions. Samples were thawed immediately prior to analysis, and absorbance was measured using a Multiskan Sky microplate spectrophotometer (Thermo Fisher Scientific, Waltham, MA, USA).

### 4.3. In Vitro Induction of Hyperosmolar Stress

To investigate the response of CCL-20.2 cells to hyperosmolar stress, the cells were cultured with 5% BCS, CBS_low_, or CBS_high_ for 24 h before damage induction. Hyperosmolar stress was induced by adding sodium chloride (NaCl; 479687; Carlo Erba Reagents, Cornaredo, Milan, Italy) to serum-free medium at a concentration of 450 mOsm/L for 3 h. At the end of the treatment, cell morphology, viability, and expression levels of TRPV1 and HLA-DR were evaluated.

### 4.4. Tropomyosin Receptor Kinase (Trk) Inhibition

To investigate the contribution of BDNF to cell response under hyperosmolar stress, the TrkB of BDNF was inhibited. To this aim, CCL-20.2 cells were pre-treated with 200 nM of K252a (K1639, CAS number: 99533-80-9; Merck Group, Darmstadt, Germany) for 1 h. The experimental design, dose, and treatment duration with K252a were established according to our previous study [19]. The cells were regularly cultured with BCS, CBS_low_, and CBS_high_ at 5% for 24 h, exposed to NaCl at 450 mOsm/L for 3 h, and analyzed in terms of cell viability and gene expression as described above.

### 4.5. Cell Viability Assay

Cell viability after exposure to NaCl and CBS treatment was determined in CCL-20.2 cells using the MTT assay (3-(4,5-Dimethylthiazol-2-yl)-2,5-Diphenyltetrazolium Bromide, Thermo Fisher Scientific, Waltham, MA, USA), following the manufacturer’s instructions. Briefly, cells were seeded in 96-well plates at 5 × 10^3^ cells/well and treated according to the experimental plan. The absorbance was measured at an optical density (O.D.) of 570 nm using a microplate reader. Data were normalized to untreated controls for each serum type.

### 4.6. RNA Extraction and Real-Time PCR

Total RNA was extracted from CCL-20.2 cells with TRIreagent (TRIzol, Thermo Fisher Scientific, Waltham, MA, USA) according to the manufacturer’s instructions. RNA concentration and quality were evaluated by a Nanodrop spectrophotometer, and Reverse Transcription was carried out using one μg of total RNA in a 20 µL reaction volume using an iScript cDNA synthesis kit (BioRad Laboratories, Hercules, CA, USA). Real-time PCR was performed using a CFX Connect Real Time PCR Detection System (BioRad Laboratories, Hercules, CA, USA) with SYBR Green chemistry (Sso Advanced TM Universal Sybr Green Supermix; BioRad Laboratories, Hercules, CA, USA). Primers sequences (Merck Group, Darmstadt, Germany) were designed with the NCBI BLAST tool (https://www.ncbi.nlm.nih.gov/tools/primer-blast/, accessed on 25 January 2026) (Table 1). Glyceraldehyde 3-phosphate dehydrogenase (*GAPDH*) was used as an endogenous control. Each assay was performed in triplicate. For gene expression analysis, the comparative 2^−ΔΔCt^ method was used [29], and results were expressed as fold changes relative to controls (cells cultured with BCS/CBS_low_/CBS_high_ vs. respective NaCl condition).

### 4.7. Immunofluorescence

Immunofluorescence was performed to investigate HLA-DR and TRPV1 expression in CCL-20.2 exposed to NaCl. To this aim, cells were seeded 3 × 10^4^/well on 18 × 18 mm glass slides, cultured with BCS/CBS_low_/CBS_high_ for 24 h, and exposed to NaCl for 3 h. After hyperosmolar stress induction, cells were fixed with 2% paraformaldehyde in PBS for 10 min, washed with PBS, and incubated with BSA 1% for aspecific sites blocking for 30 min at room temperature. The slides were then incubated overnight at 4 °C with anti-HLA-DR (1:50, clone L243, BioLegend, San Diego, CA, USA) and anti-TRPV1 (1:100, Thermo Fisher Scientific, Waltham, MA, USA). The following day, the samples were washed with PBS and incubated with anti-rabbit Alexa Fluor 546 (1:250, Thermo Fisher Scientific, Waltham, MA, USA) secondary antibody in 1% BSA/PBS for 1 h at 37 °C in the dark. After washing with PBS, nuclei were counterstained with 4′,6-diamidino-2-phenylindole, dihydrochloride (DAPI, Thermo Fisher Scientific, Waltham, MA, USA). Images were acquired using a Leica DMI4000 B inverted fluorescence microscope (Leica Microsystems, Wetzlar, Germany). Quantification of HLA-DR-positive cells was performed on digitized images randomly acquired at 20× magnification, and a minimum of three fields were examined for each sample. The results were expressed as the ratio of positive cells to total cells.

### 4.8. Trpv-1 Detection by Countess 3 FL

TRPV1 expression was further analyzed using Countess 3 FL (Thermo Fisher Scientific, Waltham, MA, USA), a benchtop instrument used for automatic cell counting and equipped with EVOs light cubes for fluorescence detection. Conjunctival cells exposed to hyperosmolar stress were fixed with 2% paraformaldehyde in PBS for 10 min, washed with PBS, stained with anti-TRPV1 antibody and anti-rabbit AlexaFluor 546 secondary antibody, and analyzed with Countess, following the manufacturer’s instructions. As previously published by our group [21], gating based on cell size ranging between 9 and 30 mm was established for each analysis. The results are expressed as mean ± SD of three measurements.

### 4.9. Statistical Analysis

Each experiment was performed in at least three biological and technical replicates. Data are expressed as the mean ± SD. Graphs and statistical analyses were performed using GraphPad Prism 10.0 software. Ordinary one-way and two-way analyses of variance (ANOVA) followed by Tukey’s test were applied to allow multiple comparisons between the three main experimental groups: BCS, CBS_low_, and CBS_high_. Results were considered statistically significant at a 95% confidence level (*p* < 0.05).

## 5. Conclusions

In conclusion, our findings demonstrate that CBS exerts both anti-inflammatory and antinociceptive effects on conjunctival epithelial cells exposed to hyperosmotic stress, highlighting its dual action on the neuro-immune interface of the ocular surface. These results support the potential of CBS as a biologically active treatment offering a promising translational approach for managing ocular surface inflammation and neuropathic pain.

## Figures and Tables

**Table 1:** Sequences of primers used for Real Time PCR.

Primer	Sequence
*GAPDH FWD*	AATGGGCAGCCGTTAGGAAA
*GAPDH REV*	AGGAGAAATCGGGCCAGCTA
*HLA-DR FWD*	GGGTCTGGTGGGCATCATTA
*HLA-DR REV*	CCATCACCTCCATGTGCCTT
*TRPV-1 FWD*	GGGTTTGGTTGGACTGGGAC
*TRPV-1 REV*	TGCAACCTGTTAGCCGGAG

*GAPDH*: Glyceraldehyde 3-phosphate dehydrogenase; *HLA-DR*: Major Histocompatibility Complex Class II (MHC II); *TRPV-1*: Transient Receptor Potential Vanilloid-1.

## Data Availability

The original contributions presented in this study are included in the article. Further inquiries can be directed to the corresponding author.

## Abbreviations

ATCC	American Type Culture Collection
BCS	Bovine Calf Serum
BDNF	Brain-Derived Neurotrophic Factor
BSA	Bovine Serum Albumin
CBS	Cord Blood Serum
CBShigh	Cord Blood Serum high BDNF
CBSlow	Cord Blood Serum low BDNF
CMV	Cytomegalovirus
CPD	Citrate-phosphate-dextrose
DED	Dry Eye Disease
EDHO	Eye Drops of Human Origin
EGF	Epidermal Growth Factor
FACT	Foundation for the Accreditation of Cellular Therapy
GAPDH	Glyceraldehyde 3-phosphate dehydrogenase
HBV	Hepatitis B Virus
HCEC	Human Conjunctival Epithelial Cells
HCV	Hepatitis C Virus
HIV	Human Immunodeficiency Virus
HLA-DR	Major Histocompatibility Complex Class II (MHC II)
HTLV-I/II	Human T-lymphotropic Virus I/II
IGF-1	Insulin-Like Growth Factor-1
IL-1β	Interleukin -1beta
IL-6	Interleukin-6
MTT	(3-(4,5-Dimethylthiazol-2-yl)-2,5-Diphenyltetrazolium Bromide
NCP	Neuropathic Corneal Pain
NGF	Nerve Growth Factor
NTFs	Neurotrophic Growth Factors
O.D.	Optical Density
PBS	Phosphate-Buffered Saline
SD	Standard Deviation
TGF-β	Transforming Growth Factor-beta
TNF-α	Tumor Necrosis Factor alpha
TrkB	Tropomyosin Receptor Kinase B
TRPV-1	Transient Receptor Potential Vanilloid 1
VEGF	Vascular Endothelial Growth Factor
